# Analysis by Light, Scanning, and Transmission Microscopy of the Intima Synovial of the Temporomandibular Joint of Human Fetuses during the Development

**DOI:** 10.1155/2014/732720

**Published:** 2014-01-12

**Authors:** Carlos Sabu Alvez, Luis Otavio Carvalho de Moraes, Sergio R. Marques, Roberto C. Tedesco, Leandro J. C. Harb, Jose F. Rodríguez-Vázquez, Jose R. Mérida-Velasco, Luis Garcia Alonso

**Affiliations:** ^1^Departamento de Morfologia e Genética, Universidade Federal de São Paulo, 04023-900 São Paulo, SP, Brazil; ^2^Departamento de Morfologia, Universidade Federal de Santa Maria, 97105-900 Santa Maria, RS, Brazil; ^3^Departamento de Anatomía y Embriología II, Universidad Complutense of Madrid, 28040 Madrid, Spain

## Abstract

*Objective*. To characterize morphologically and ultrastructurally using light microscopy, the scanning electron microscopy and transmission electron microscopy the intima synovial of the temporomandibular joint (TMJ) of human fetuses between the 10th and the 38th week of development. *Materials and Methods*. The TMJ was dissected bilaterally in 37 human fetuses belonging to the Institute of Embryology of the University Complutense of Madrid and of the Federal University of São Paulo. *Results*. The outcome by light microscopy showed the morphology of the TMJ and that the formation of inferior joint cavity precedes the superior joint cavity and the presence of blood vessels in the synovial. *Conclusion*. By scanning and transmission electron microscopy we observed the presence of two well-defined cell types in the intima layer of synovial of the TMJ of human fetuses, macrophage-like type A cell and fibroblast-like type B cell, and the presence of the a third cell type, defined by the name of intermediate lining cell in the intima layer of the synovial.

## 1. Introduction

The temporomandibular joint (TMJ) is a specialized synovial joint essential for the function of the mammalian jaw. The main components of the TMJ are the mandibular condyle, the mandibular fossa of the temporal bone, the articular disc with collagen fibers interposed with between them, and a synovial [1–5].

Morphologically, the synovial consists of two layers: one cellular intima layer and another for support, the vascular subintima layer, which combines with the articular capsule. The intima layer consists of cells within an amorphous and fiber-free matrix whose width varies approximately from 1 to 4 cells. The subintima layer consists of loose and vascularized connective tissue, with spread out fibroblasts, macrophages, mastocytes, adipose cells, and some elastic fibers that prevent the pleating of the [6–12].

The intima layer has cells with phagocytic ability called macrophage-like type A cell. This same layer also has cells called fibroblast-like B cells, which synthesize proteins, glycoproteins, and proteoglycans [9, 10, 13–16].

There is also a third type of cells not entirely studied yet called intermediate lining cell [6, 17–20]. Among these three cell types there are spaces filled with not very fibrous extracellular matrix and macrophage ground substance.

The synovial fluid is formed in the synovial by blood vessel plasma in the subintima layer and travels to the articular space. As it crosses the intima layer, new elements secreted by fibroblast-like type B cells join in and the fluid becomes the so-called synovial fluid. The synovial fluid is constantly renewed and, when it crosses back the intima layer, it is affected by the macrophage-like type A cells that phagocyte the proteic and glycidic part of the fluid. The remaining plasma reaches the subintima layer and returns to the circulation through the lymphatic and venous capillaries therein. The synovial has high metabolic activity and a considerable degeneration power [11, 21, 22].

The morphological and ultrastructural understanding of the TMJ along the process of organogenesis and fetal development is important not only to better understand the embryological steps that culminate in its anatomic constitution but also to elucidate intrinsic mechanisms that may be involved in morbid and pathological situations that may occur after birth.

Therefore, the objective of this study is to characterize morphologically and ultrastructurally, using light microscopy, scanning electron microscopy, and transmission electron microscopy, the synovial of the TMJ of human fetuses between the 10th and the 38th week of development.

## 2. Material and Methods 

We studied 37 human fetuses belonging to the Institute of Embryology of the University Complutense of Madrid and of the Federal University of São Paulo. The specimens ranged from 52 mm to 335 mm greatest length (GL) and the ages were three fetuses of 10th weeks, two of 11th, seven of 12th, three of 13th, three of 14th, one of 15th, one of 16th, one of 17th, two of 18th, three of 24th, four of 28th, two of 30th, three of 32th, and one each at 36th and 38th weeks of development. The parameters used to determine postconception age were the GL, external and internal criteria [23, 24]. All specimens are from ectopic pregnancies or spontaneous abortions, and none of the material indicated possible malformation.

All fetuses were fixed in 10% formalin and separated into groups which were studied using light microscopy, scanning electron microscopy and transmission electron microscopy.

The twenty-three samples between 10th and 18th weeks were selected for light microscopy. The samples were decalcified in EDTA for 21 days and rinsed with tap water for 10 minutes. Next, they were fixed in 10% formaldehyde for 24 hours. Later, the following dehydration sequence was done: 50% ethanol for 24 hours, 70% ethanol twice at every six hours, and absolute ethanol twice. Finally, the samples were made translucent by a 2-hour treatment with xylol and fixed in paraffin. Semiserial, frontal and sagittal 4 *μ*m cuts of the TMJ were made by the microtome Leica model RM2035. The samples were then stained with hematoxylin-eosin (HE) and the widely used Nylceo Marques de Castro trichrome stain [25] and covered with a coverslip.

The six samples at 24th and 32th weeks were selected for the scanning electron microscopy study. The TMJ was removed from the 10% formalin solution and rinsed in a 0.1 M cacodylate buffer with pH 7.2. All the samples were cryofractured in liquid nitrogen and then treated with decreasing concentrations of sodium hypochlorite (from 2% to 0.2%). After dehydration using a 50%, 70%, 90%, and absolute ethanol series, the specimens were dried by the critical point method using carbon dioxide in the device Balzers CPD010 and placed in the specimen holder. The samples were coated in gold by the device Balzers SDC Sputter Coater and examined by the scanning electron microscope Jeol 5300.

The eight samples at 28th, 30th, 36^th^, and 38th weeks were prepared by a protocol of four days for the transmission electron microscope imaging. On the first day, the samples were placed in a fixating solution consisting of 2.5% glutaraldehyde and 2.0% formaldehyde and rinsed with a 0.1 M sodium cacodylate buffer with a pH of 7.2.

On the second day, the samples were placed in a 2.0% solution of osmium tetroxide together with the abovementioned 0.1 M buffer solution for two hours. Next they were rinsed again by a 0.1 M buffer solution repeated 3 times at 30-minute intervals. Dehydration was done by a series of ethanol solutions at 70% and 90% for two hours each and absolute ethanol for one hour. Finally, the material was allowed to spend the night in absolute ethanol.

On the third day, the samples were soaked in the resin Epon in the proportion of 2 : 1 for two and a half hours and allowed to spend the night in propylene oxide and Epon in the proportion of 3 : 1.

On the last day, the material was placed in concentrated Epon resin for two hours followed by a vacuum chamber also for two hours. Finally, the material was made into blocks and placed in an incubator for 48 hours at 60°C. The material was then imaged with transmission electron microscopy.

The study was approved by the Ethics Committee of the Faculty of Medicine of the University Complutense of Madrid and the Federal University of São Paulo Research Ethics Committee (CEP: 0917-04).

## 3. Results

**Figure 1:**
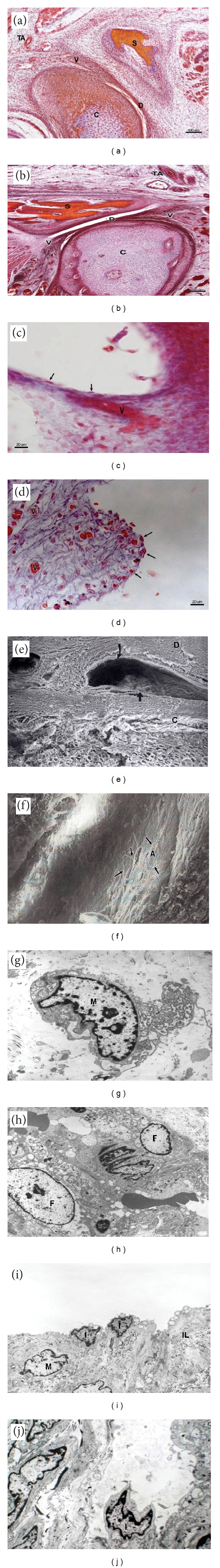
(a) human fetus (55 mm GL; 10th week of development). Frontal section. Hematoxylin-eosin. The inferior joint cavity is between the articular disc (D) and the mandibular condyle (C). Squamous part of the temporal bone (S); blood vessel (V); superficial temporal artery (TA). Bar = 1000 m. (b) Human fetus (95 mm GL; 12th week of development). Frontal section. Hematoxilyn-eosin. The superior and inferior joint cavities are visible. Articular disc (D). Blood vessel (V); squamous part of temporal bone (S); mandibular condyle (C); superficial temporal artery (TA). Bar = 2000 m. (c) Human fetus (125 mm GL; 14th week of development). Sagittal section. Hematoxilyn-eosin. Blood vessel (V) and blood cells (arrows) are visible at the synovial. Bar = 200 m. (d) human fetus (175 mm GL; 18th week of development). Sagittal section. Trichome Nylceo Marques de Castro. Section of the posterior region of the superior joint cavity. Blood vessel (V); lining cells are visible at the synovial (arrows). Bar = 200 m. (e) Human fetus (233 mm GL; 24th week of development). SEM (×198). The inferior joint cavity on the fibrous portion (C) of the condyle, the articular disc (D), and the synovial (arrows) is observed. (f) Human fetus (300 mm GL; 32th week of development). SEM (×920). Synovial lining cell (A) with a smooth surface formed by polygonal cells with clear limits (arrows). ( g) Human fetus (265-mm GL; 28th week of development). TEM (×6000). Macrophage-like type A cell (M). ( h) Human fetus (285 mm GL; 30th week of development). TEM (×3000). Fibroblast-like type B cell (F). (i) Human fetus (325 mm GL; 36th week of development). TEM (×3000). The intima layer (IL) of synovial is been formed with intermediate lining cells (I) and macrophage-like type A cell (M). (j) Human fetus (335 mm GL; 38th week of development). TEM (×3000). Intermediate lining cell (I) protruding in the intima layer of the synovial is visible.

In specimens of 10th weeks of development shows the outline of the TMJ articular disc disposed between the mandibular condyle and the squamous part of the temporal bone. An apparent tissue separation was clearly recognizable between the mandibular condyle and articular disc, indicating the commencement of formation of the inferior articular cavity (Figure 1(a)). In specimens at 12th weeks of development, the superior joint cavity is evident. Some vessels are observed in relation anterior and posterior region of the articular disc (Figure 1(b)).

In specimens at 14th weeks of development is seen the outline of synovial in the posterior region of the inferior joint cavity of the TMJ (Figure 1(c)). The intima layer of the synovial is composed of synoviocytes. In specimens at 18th weeks of development shows the superior joint cavity in the posterior region of the TMJ. It also shows the intima layer of the synovial, lining cells, and possible blood vessels in the subintima layer (Figure 1(d)).

Meantime scanning electron microscopy shows the posterior region of the inferior joint cavity limited by the synovial. It is possible to observe the synovial lining in the intima layer with the smooth surface formed by polygonal cells and clear borders (Figures 1(e) and 1(f)).

The specimens analyzed by transmission electron microscopy show in the intima layer three cell types: one with more dense chromatin disposed on the inner side of the nuclear and rare nucleoli, with presence of a complex Golgi apparatus, vesicles and vacuoles dispersed in the cytoplasm, scarce rough endoplasmatic reticulum, corresponding to macrophage-like type A cells; another type of cell characterized by the presence of rougher endoplasmic reticulum and numerous nucleoli, corresponding to fibroblast-like type B cells; and the third cell type of cell with characteristic similar to the macrophage-like type A, although these cells have more intensely stained nuclei and smaller size. Additionally, these cells are very much on the surface of the intima layer, corresponding to intermediate lining cells (Figures 1(g) to 1(j)).

## 4. Discussion

In agreement with [26], in human fetuses, the process of joint cavitation is not synchronic since organization of the inferior joint cavity precedes that of the superior one. The inferior joint cavity begins at the end of week 9th, and the superior joint cavity begins during the 11th week of development. Moreover, In TMJ development, the blood vessels have been reported to run posteroanteriorly on the inferior surface of the articular disc at the early stages of joint cavity formation [1, 2]. In particular, Ohnuki [2] found the disintegration of the blood vessels as merged with the forming inferior joint cavity, suggesting that blood vessels serve as a partition between the articular disc and the condyle to produce a space for the inferior joint cavity in the mesenchymal tissue. Ikeda et al. [21] reported communication between the inferior articular cavity and the surrounding blood vessels during the development of rat TMJ. We have stated that specimens at 10th weeks of development showed a clear inferior articular cavity and some branch vessels located peripherally. In specimens at 12th weeks of development showed a clear superior joint cavity and vessels in the anterior and posterior region of the articular disc. These findings could suggest the possibility that the vascularization around the TMJ is closely related to the articular cavity formation and therefore with the formation of synovial.

Our findings regarding the synovial are in agreement with those of other authors [9, 19, 26]. Hence, in the study fetuses, the internal surface of the articular capsule is lined by a synovial that coats the intra-articular structures, except for the articular cartilage present in the articular tubercle, the roof of the mandibular fossa and the mandibular condyle. In the articular disc, the covers only short segments of the anterior and posterior ends. The synovial villi are finger-like projections in the anterior and posterior borders of the joint in order to accommodate the movements of the articular capsule [12, 27, 28].

Transmission electron microscopy evidenced the presence of an intermediate layer of lining cells on the superior and posterior regions of the superior joint cavity and synovial. This layer consists of flat cells and intensely stained nuclei and corresponds to an unusual finding with regard to the pertinent literature.

This layer of flat cells, probably mesenchymal, lines the superior and inferior joint cavity as a continuation of the synovial. Such lining stratus is similar to a mesothelium and may be justified as originating from the mesenchyme. Inside the mesenchyme, small cracks gradually coalesce to form the final configuration of the articular cavities [28].

As the articular dynamics establishes itself, the cell lining is likely reabsorbed. Since in adults this layer is not mentioned in the literature, this lining may be indeed reabsorbed but probably during postnatal development.

Some authors studied the histological aspects of this region in detail but did not cite or describe this layer of intermediate lining cells [9, 10, 13–16, 29, 30]. Meanwhile Barland et al., Fell et al., Horký, and Nagai et al. [6, 17, 18, 20] studied this region and, like the present study, noted the presence of these intermediate lining cells.

Classically, the synovial consists of two layers: an intima layer formed by fibroblast-like type B cells, similar to fibroblasts that synthesize proteins, glycoproteins and proteoglycans, and macrophage-like type B cells, similar to macrophages (and with phagocytic capacity) and another support layer under the first one called subintima (with plenty of blood and lymphatic vessels) that blends in with the articular capsule [9, 29].

The observation of this unusual morphological characteristic made us consider the possibility of studying it minutely using transmission electron microscopy. And the results confirm the light microscopy and scanning electron microscopy findings indicating the presence of intermediate lining cells in the intima layer of the synovial.

Our results by the transmission electron microscopy study show the macrophage-like type A and fibroblast-like type B cells of the intima layer of the synovial. The first type has been named by some authors as synovial macrophages, M cells, A cells (absorptive), and V cells (vacuoles) while the second type, fibroblast-like type B cells, has already been called synovioblasts, F cells, S cells (secretory), and RE cells (endoplasmic reticulum) [10]. The classic proposition of [6] nomenclature names these cells as types A and B, respectively. This review about synoviocytes uses the most currently accepted nomenclature.

The macrophage-like type A cells are characterized by the presence of a complex Golgi apparatus, vesicles, and vacuoles dispersed in the cytoplasm, scarce rough endoplasmic reticulum, and a dense pattern of nuclear chromatin and rare nucleoli. On the other hand, the fibroblast-like type B cells are characterized by the presence of rougher endoplasmic reticulum and numerous nucleoli [8].

The profile of the intermediate lining cells of the present sample has ultrastructural features that indicate a relationship with the macrophage-like type A cells. This leads to the following question: if the intermediate lining cells are in an initial phase of differentiation, will they migrate to the inside of the intima layer, or are they migrating from inside the intima layer to the surface?

An explanation that could justify the presence of lining cells in the synovial of the TMJ of fetuses and not in those of fully formed individuals is what occurs with the Nasmyth membrane, for example. This membrane corresponds to a dental cuticle or a fine membrane on the surface of the dental enamel formed by reduced enamel epithelium and the basal lamina [30] and it gradually detaches after the eruption of the tooth. This membrane might be the remains of the enamel organ [31]. Likewise, given the similarities between the fibroblast-like type B cells and the intermediate lining cells, one may suppose that the latter are the remains of fibroblast-like type B cell formation and that they could serve as lining, just like the Nasmyth membrane, and detach after birth once the TMJ started being used.

We can compare the intermediate lining cells with the classic macrophage-like type A cell, capable of phagocytosis. The first ones have a more intensely stained nuclei and a smaller size. Additionally, the intermediate lining cells are very much on the surface of the intima layer while the macrophage-like type A cells are deeper down in the layer. Their ultra-structure indicates that they are distinct cell types.

## 5. Conclusions

In summary, our study shows that there are two well-defined cell types in the intima layer of synovial of the TMJ of human fetuses: macrophage-like type A cell and fibroblast-like type B cell, and it indicates the presence of the a third cell type, defined by the name of intermediate lining cell, in the intima layer of the synovial.
